# Significance of Ionic Character Induced by Ga-Doped γ-Al_2_O_3_ on Polyethylene Degradation to the Precursors of Gasoline and Diesel Oil with a Trace Amount of Wax

**DOI:** 10.3390/nano12183122

**Published:** 2022-09-09

**Authors:** Syed Kamran Haider, Amol Uttam Pawar, Don Keun Lee, Young Soo Kang

**Affiliations:** 1 Department of Chemistry, Sogang University, 35, Baekbeomro, Mapogu, Seoul 04107, Korea; 2 Environmental and Climate Technology, Korea Institute of Energy Technology, Naju-si 58219, Korea

**Keywords:** polyethylene degradation, Ga-doped γ-Al_2_O_3_, carboxylic acid, C-C and C-H bonds, low temperature

## Abstract

Polyethylene degradation has a significant ecological impact but is also economically beneficial because it generates fuels and useful chemical products. Our study mainly describes the cleavage of C-C and C-H bonds when polyethylene (dispersed in 1-octadecene) was low-temperature heat-treated in two steps, at 180 and 250 °C, for 24 h for each step. Finally, it was converted to a mixture of the precursors of gasoline and diesel oil with a trace amount of wax. A series of reactions resulted in cracking, dehydrogenation and oxidation, hence producing polycarboxylic acids and saturated and unsaturated hydrocarbons. ESI-MS analysis revealed that mixed oil consisted of low carbon number hydrocarbons and their derivatives of carboxylic acids, with the carbon number ranging from C-6 to C-18. In the trace amount of wax, complicated carboxylic acids and hydrocarbons with carbon number C-22 to C-58 were also identified. FT-IR analysis further confirmed the presence of carboxylic acid derivatives and double bonds in the degradation products. γ-Al_2_O_3_ nanorods effectively catalyzed the degradation process by enhancing the C-C chain length in the products. Lewis acid (Al) and Lewis base (oxygen) in the γ-Al_2_O_3_ induced ionic character of the C-C bond chain, which led to the efficient cracking of the C-C bond. Poor shielding effect, smaller atomic size and greater ionization energy made Ga a stronger Lewis acid compared to Al; hence, Ga-doped γ-Al_2_O_3_ catalyzed the degradation process even more effectively.

## 1. Introduction

Polyethylene (PE) waste has inflicted adverse impacts on land and water and is one of the most important challenges to be solved for a green and clean Earth [1]. Total global PE waste generation is expected to increase up to 900 million tonnes per year by the end of this decade [1]. It has recently been estimated that because of the huge industrial growth in China and fellow Asian countries, plastic waste generation has tripled from 2005 to 2020 [2]. 

Microplastics (1 μm–5 mm) and nanoplastics (1–1000 nm) are continues source of danger for aquatic organisms and have gained scientific and public attention worldwide [3,4]. Microplastics (MPs) may cause growth inhibition, oxidative damage and immune stress; furthermore, when accumulated in marine organisms, they may move through the food chain to the higher trophic levels (e.g., humans) [5,6]. Human consumption of MPs through water and food is estimated to range from 203 to 332 particles per person per day [7]. 

MPs can also cause soil pollution by changing the bulk density and water holding capacity and degrading organic matter in the soil, which leads to abnormal plant growth [8,9]. In addition, MPs can absorb organic pollutants and heavy metals owing to their large specific surface area [10,11]. MPs have been found in many small soil invertebrates, such as snails [12] and nematodes [13], and are transferred to the food chain [14]. Ingestion of MPs can lead to false satiation, causing biological harm such as clogging or abrasion of the digestive tract and reducing feeding rates [15]. 

Degradation of PE is not only a matter of interest because of its ecological impact, but it is also economically beneficial because it generates fuels and useful chemical products [16,17]. PE degradation can take various routes, e.g., biotic, thermal, chemical, pyrolysis or plasma degradation [18,19,20]. Thermal degradation is being used at an industrial scale to degrade PE to obtain useful organic products [21,22,23,24,25], but this process requires high temperatures (typically > 400 °C) which consume a lot of thermal energy. Although this is the most commonly used method for waste PE degradation, it can produce harmful gases and VOCs [26]. Mechanical degradation of polyethylene can also be useful but it produces microparticles that contribute to soil and water pollution. Polyethylene has been degraded to useful products by heat treatment at relatively low temperatures, though in this case, Re and Pt-based expensive catalysts have been used [27]. These expensive metal-based catalysts cannot be used on an industrial scale because even though the catalytic poisoning is minimal, the degradation process is not economically viable. Furthermore, these catalytic degradation processes are complicated and are divided into further sub-steps, e.g., dehydrogenation, olefine metathesis and hydrogenation. 

Heat treatment in an autoclave provides the extreme conditions, including high temperature and pressure, that generate the radicals [28]. Working with a heterogeneous catalyst is very useful because it is easy to remove after the reaction [29]. PE degradation by autoclave annealing can be very useful if proper solvent and low-cost heterogeneous thermo-catalysts are used. γ-Al_2_O_3_ has been used as a catalyst for the dehydrogenation of alkanes [30]. It is usually produced by calcination of boehmite (γ-AlOOH) at 300–500 °C [31,32]. Al in the oxide acts as the Lewis acid and oxygen acts as the Lewis base. Therefore, it can induce ionic character to the C-C bonds based on the oxygen defects on its surface. The degradation efficiency of γ-Al_2_O_3_ is further enhanced by Ga doping as Ga is a stronger Lewis acid compared to Al due to its distorted crystal structure and oxygen defects. 

Herein, we introduce a simple method by which polyethylene can be converted to the precursors of gasoline and diesel oil with a trace amount of wax under mild conditions in the presence of γ-Al_2_O_3_. A hydrothermal method was used to synthesize the undoped and (2%) Ga-doped γ-Al_2_O_3_. These catalysts effectively catalyzed polyethylene degradation to the precursors of gasoline and diesel oil of carbon number less than 18 with a trace amount of wax. The degradation mechanism and product analyses have been clearly elaborated. Furthermore, the role of γ-Al_2_O_3_ (undoped and Ga-doped) on the degradation process is also elaborated.

## 2. Experimental Section

### 2.1. Materials

1-octadecene (C_18_H_36_), polyethylene sheet, ethanol (C_2_H_5_OH), aluminum nitrate nonahydrate (Al(NO_3_)_3_.9H_2_O), sodium hydroxide (NaOH), gallium (III) nitrate hydrate (Ga(NO_3_)_3_.xH_2_O), methanol (CH_3_OH) and chloroform (CHCl_3_) were obtained from Sigma Aldrich (South Korea). These analytical-grade reagents were used without further purification. The Milli-Q IQ 7000 water purifying system was used to obtain deionized water.

### 2.2. Characterization 

Crystal structure analysis was performed by X-ray diffractometer (Rigaku MiniFlex) with a Cu-Kα source radiation wavelength of 0.15418 nm. TEM, TEM-EDX and HRTEM characterizations were performed on JEM-2100F by JEOL Ltd. For TEM analysis, γ-Al_2_O_3_ and PE were dispersed in ethanol and toluene, respectively. A Ni TEM grid was used for TEM and HRTEM analyses. An ESI-Iontrap Mass Spectrometer (Model: LTQ XL) by Thermo Fisher Scientific was used for the ESI-MS (Electrospray Ionization Mass Spectrometry) analysis. To estimate the functional groups in the degraded products, a Fourier Transform (FT)-IR in a Nicolet iS50 (Thermo Fisher Scientific spectrometer) with a deuterated-triglycine sulfate (DTGS) detector was used.

### 2.3. Synthesis of γ-Al_2_O_3_ Nanorods

Boehmite nanorods were synthesized by hydrothermal treatment based on the previously reported method with slight modification [33], then 2.4 g of aluminum nitrate nonahydrate (Al(NO3)3.9H_2_O) was dissolved in a mixture of 10 mL DI water and 5 mL (1 M) NaOH solution. The mixture was transferred to a 45 mL Teflon-lined autoclave and treated hydrothermally for 20 h at 200 °C. After the heat treatment, the autoclave was allowed to cool to room temperature and the white powder (g-AlOOH) was separated from the solution by centrifugation (6000 rpm/15 min). The product was washed several times with DI water and dried overnight at 80 °C under vacuum. After vacuum drying, the g-AlOOH powder was calcined at 500 °C for 3 h and γ-Al_2_O_3_ nanorods were obtained. To synthesize the Ga-doped γ-Al_2_O_3_, in another separate experiment, 0.033 g of gallium (III) nitrate hydrate (Ga(NO_3_)_3_.xH_2_O) was added to the mixture of alkaline solution of Al(NO_3_)3.9H_2_O (2.352 g). All other steps of the experiment remained constant for the synthesis of γ-Al_2_O_3_.

### 2.4. Polyethylene Degradation

In the typical closed system, 300 mg of polyethylene powder was dissolved in 50 mL of 1-octadecene in a Teflon-lined autoclave. Then, 100 mg of the γ-Al_2_O_3_ (or Ga-doped γ-Al_2_O_3_) nanorods was added to this mixture in two other separate experiments.

The mixture was heated at 180 °C for 24 h. The autoclave was then cooled to room temperature and the solid residue (wax and catalyst) was filtered and the semisolid mixture was further heated for 24 h at 250 °C. It was found that if the liquid is not filtered after the first step, and heated together with the wax (in the second step annealing), degradation of the wax is very low and can stop the flowing tube during the reaction, which suppresses the degradation reaction waste plastics. A two-step heat treatment produced a mixture of the precursors of gasoline and diesel oil with a trace amount of wax. Oil precursors were separated from the solid components (wax and catalyst) by centrifugation. To separate the wax and catalyst, a binary solvent composed of chloroform and methanol (*v/v*, 2:1) was used. Wax was dissolved in the binary solvent and the catalyst was removed by centrifugation. Oil precursors and wax were stored separately and analyzed by ESI-MS and FTIR.

## 3. Results and Discussion

This study mainly describes the role of a catalyst in the cleavage of the C-C bond in PE. Both the solute and solvent (PE and 1-octadecene) have a C-C bond and the cracking of both has been studied. At first, the γ-Al_2_O_3_ catalyst was synthesized, with the synthetic process consisting of four steps. These steps included precipitation, aluminum tri-hydroxide formation, conversion of aluminum tri-hydroxide to boehmite and, finally, the formation of γ-Al_2_O_3_ from boehmite. 

When the reactants were taken into the autoclave, Na^+^ and OH^−^ ions were formed in the solution. These ions were converted from Al(NO_3_)_3_.9H_2_O to Al(OH)_3_. At an elevated temperature, the following reaction occurred in the hydrothermal conditions and boehmite was produced. 

**2Al(OH)_3_ → 2AlOOH + 2H_2_O**

The produced boehmite was washed to remove impurities then converted to γ-Al_2_O_3_ by annealing at 500 °C. Other chemical methods (e.g., co-precipitation [34,35], salt spray method [36], sole gel method [37]) can operate at relatively lower temperatures but they are not useful for producing particles with the controlled size and morphology. A detailed characterization of γ-Al_2_O_3_ is described in the Supplementary Information (Figure S1) but here we will only discuss the structure of the Ga-doped γ-Al_2_O_3_.

**Figure 1:**
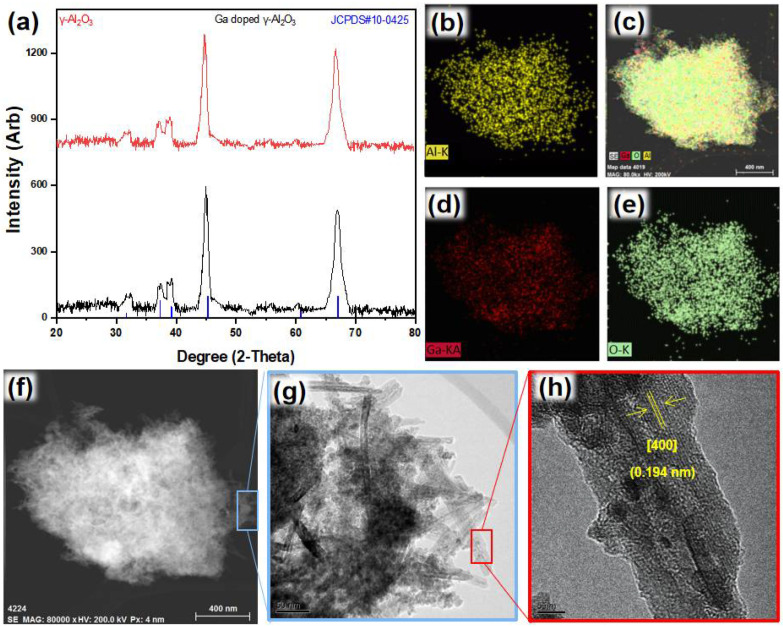
(**a**) XRD, (**b**–**e**) TEM-EDS, (**f**,**g**) TEM and (**h**) HRTEM of Ga-substituted γ-Al_2_O_3_.

Nanorods of the Ga-doped γ-Al_2_O_3,_ with an average length of 250 nm, were produced (Figure 1g). The average width of the rods was determined as 22 nm. XRD patterns (Figure 1a) confirmed the formation of the γ-Al_2_O_3_ phase (JCPDS #10-0425). A slight peak shift in the right side was observed after Ga doping in the γ-Al_2_O_3_, which was most probably because of the reduction in the d-spacing owing to the smaller atomic radius of Ga compared to Al. HRTEM analysis identified the presence of [400] facet with a d-spacing value of 0.194 nm (Figure 1h). The recorded value of d-spacing is slightly less than the standard d-spacing value (0.197 nm) of [400] facet of γ-Al_2_O_3,_ which indicates the contraction of the d-spacing because of Ga doping and crystal structure distortion of the single crystalline g-Al_2_O_3_ to induce ionic character. The reduction in d-spacing may refer to the substitution of an atom with another atom of smaller atomic radius [38,39,40]. This leads to the distortion of the crystal structure in addition to the oxygen defects. TEM-EDS (Figure 1b–e) also confirmed the homogeneous even distribution of Ga in the γ-Al_2_O_3_.

**Figure 2:**
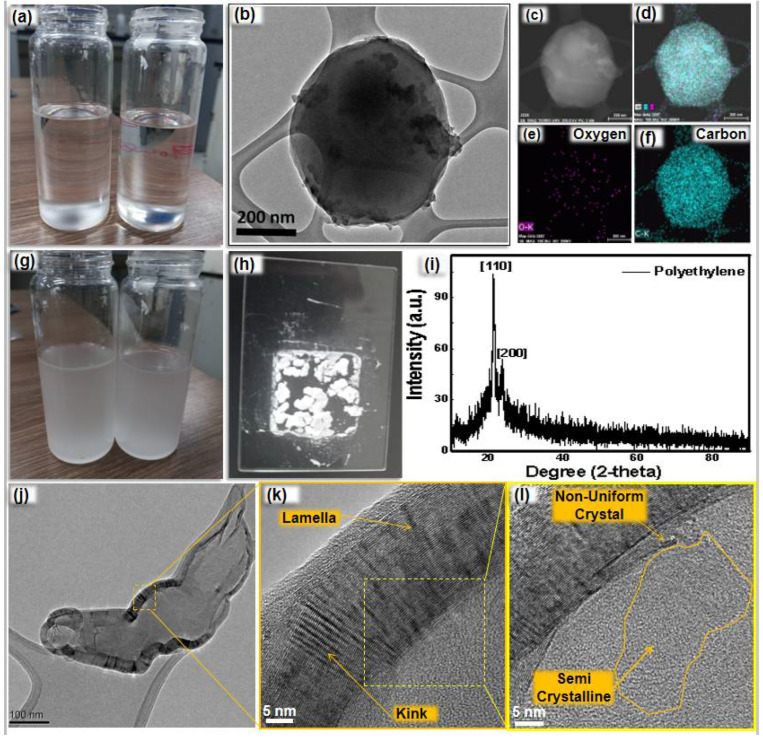
(**a**) Solution of PE in toluene at 105 °C; (**b**) TEM and (**c**–**f**) TEM-EDS of PE particles; (**g**) PE in toluene at room temperature; (**h**) semi-crystalline PE powder after removal of solvent; (**i**) XRD patterns, (**j**) TEM and (**k,l**) HRTEM of semi-crystalline PE.

A relative quantity of the PE versus the catalyst quantity critically affected the degradation process. It was observed that when the polyethylene concentration increased above 300 mg (in 50 mL of 1-octadecene), it was partially degraded. Leftover polyethylene was found as a layer at the bottom of the autoclave. For the analysis, the leftover layer was separated, dried and dissolved in toluene at 105 °C (Figure 2a). TEM and TEM-EDS images for the dissolved PE particles are shown in Figure 2b–f, respectively. It was found that the PE solution was solidified as gel at room temperature (Figure 2g). This gel was kept at room temperature for one week and the solvent (toluene) was completely dried, leaving the white semi-crystalline PE powder (Figure 2h). XRD (Figure 2i), TEM (Figure 2j) and HRTEM (Figure 2k,l) analyses of the semi-crystalline PE were performed. [110] and [200] crystal facets of PE were detected in the XRD patterns. HRTEM analysis also confirmed the presence of kinks and lamella structures. 

**Figure 3:**
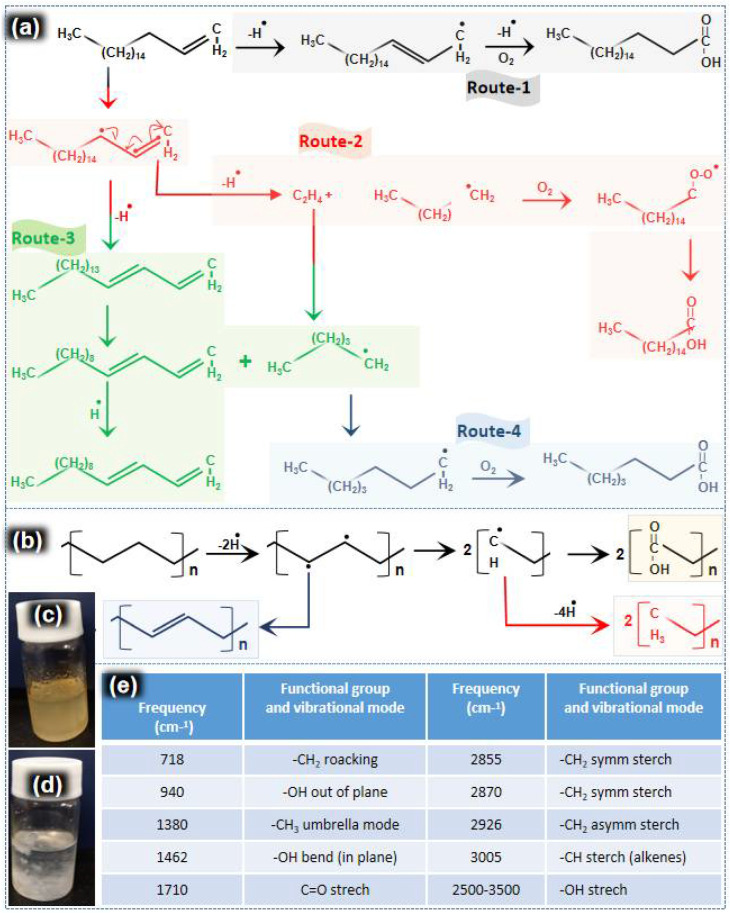
(**a**) Proposed mechanism of degradation of 1-octadecene, (**b**) PE, (**c**) oil precursors, (**d**) wax (suspended in acetone) produced by the degradation process and (**e**) FTIR analysis of the final product (oil precursors with a trace amount of wax).

Heat treatment of PE in 1-octadecene degraded both PE and 1-octadecene. During heat treatment at high temperature and pressure, one hydrogen radical was removed from 1-octadecene and an allyl radical was formed. Based on the mechanism pathway, it was assumed that the 1-octadecene ally radical was degraded in four different routes as shown in routes 1, 2, 3 and 4 of Figure 3a. These four routes mainly deal with the formation of alkenes and carboxylic acids, with their mechanisms well established in the literature. The mechanisms shown in Figure 3 are also deduced from those studies [41,42].

Following route-1, a hydrogen radical was added to the 1-octadecene allyl radical and further oxidation led to the formation of stearic acid. Degradation by route-2 shows that one more hydrogen radical was removed and C_18_H_34_ was produced. Further dehydrogenation and oxidation of C_18_H_34_ led to the formation of palmitic acid. Following route-3, the vinyl group was removed from the 1-octadecene allyl radical which eventually produced C_16_H_31_ radical. Further dehydrogenation finally converted it to C_16_H_30_ (hexadecadiene). Degradation by route-4 led to the formation of valeric acid. In conclusion, 1-octadecene degradation produced a mixture of products mainly consisting of palmitic acid, stearic acid, hexadecadiene and valeric acid.

**Figure 4:**
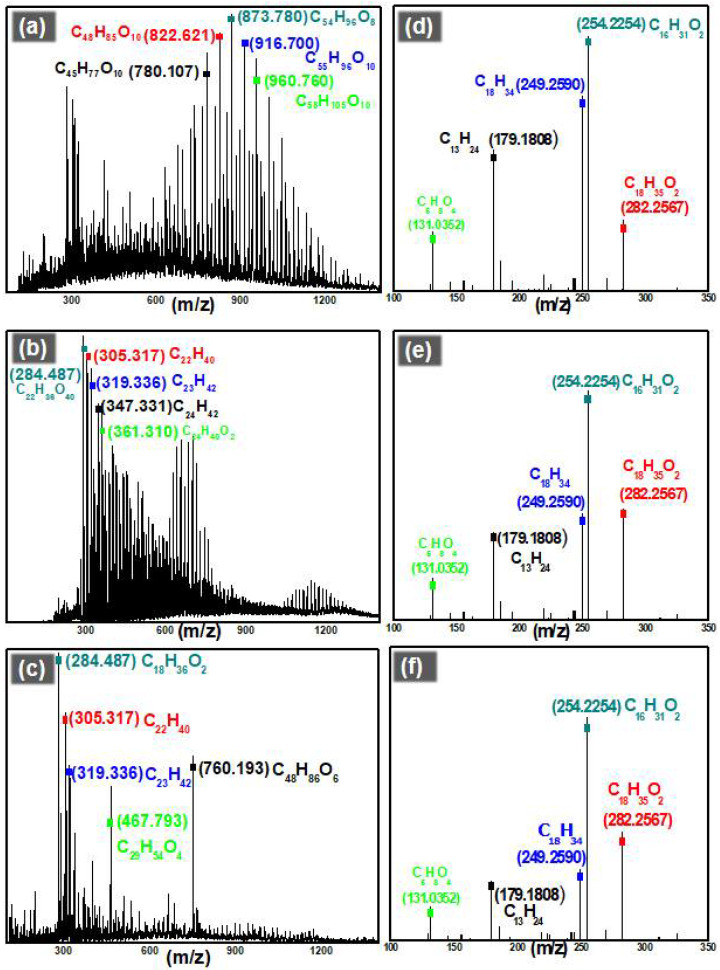
ESI-MS analysis of the wax produced by degradation of PE (**a**) without catalyst, (**b**) catalyzed by γ-Al_2_O_3_ and (**c**) catalyzed by Ga-doped γ-Al_2_O_3_. ESI-MS analysis of the oil precursors produced by degradation of 1-octadecene (**d**) without catalyst, (**e**) catalyzed by γ-Al_2_O_3_ and (**f**) catalyzed by Ga-doped γ-Al_2_O_3_.

The mechanism for degradation of PE is also proposed in Figure 3b. Hydrogen was firstly removed from the PE chain. The unstable C-C bond, after the bi-radical formation (Figure 3b), can either break or form a double bond. After cracking of the C-C bond, two chains with carbon radicals at their ends were generated. These two chains were further oxidized to form carboxylic acid. Hence, a trace amount of wax consisting of polycarboxylic acids and unsaturated hydrocarbon was obtained (Figure 3c,d). The degradation products were examined by FTIR analysis (Figure 3e) and ESI-MS (Figure 4a–f).

For FTIR analysis, a mixture of oil precursors and wax was dissolved in the binary solution of chloroform and methanol (*v/v*, 2:1). Binary solvent was run as a baseline to subtract the solvent peaks (Figure S2). FTIR further confirmed the presence of the functional groups proposed in Figure 3a,b. For CH_2_, a symmetric stretch (2855, 2870 cm^−1^) and rocking vibration (718 cm^−1^) appeared. A C-H stretching peak for alkenes was also observed at 3005 cm^−1^. A broad peak for OH stretching was observed at 2500–3500 cm^−1^. Furthermore, in-plane (940 cm^−1^) and out-of-plane (1462 cm^−1^) OH bands were also recorded. The presence of OH groups and the huge peak for carbonyl stretching (C=O) (1710 cm^−1^) indicates the formation of carboxylic acid. 

For the ESI-MS analysis of oil precursors, the m/z scan range was selected as 90–600 (Figures S3–S5). However, for wax, an m/z scan was performed between the range of 100–2000 (Figures S6–S8). In the case of oil precursors, analyte was ionized by the removal of protons and the value of m/z was actually [M − H^+^], as M is the molecular mass of the degradation product. However, in the case of wax, analyte was positively ionized by the addition of protons and the value of m/z was [M + H^+^]. ESI-MS analysis revealed that oil precursors consisted of a mixture of hydrocarbons and carboxylic acids. The carbon number of the compound in the mixture varied from C-6 to C-18. Figure 4d shows the composition of oil precursors produced without any catalyst. Figure 4e,f reveal the composition of oil precursors produced by γ-Al_2_O_3_ and the Ga-doped γ-Al_2_O_3_ catalyzed degradation, respectively. Furthermore, it was confirmed by ESI-MS that wax obtained by degradation of PE consisted of complicated carboxylic acids and hydrocarbons with carbon number of C-22 to > C-58. Figure 4a–c show the composition of wax produced by PE degradation. A list of the main products obtained during the degradation process is provided as Table S1 in the supporting information.

**Figure 5:**
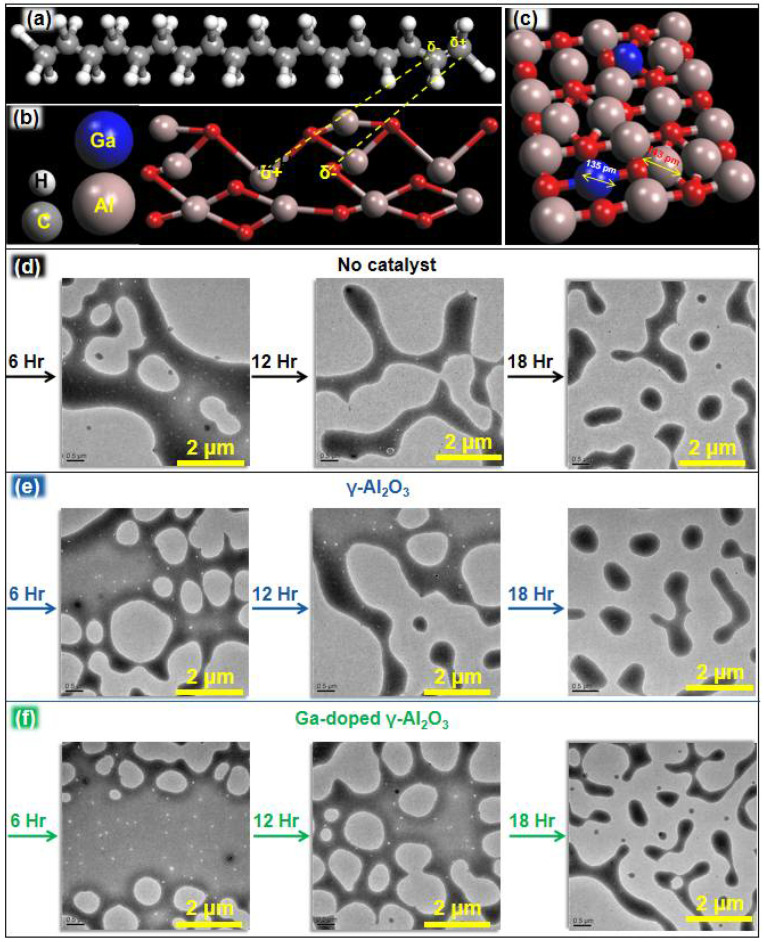
(**a**) 1-octadecene, (**b**) attachment of 1-octadecene on γ-Al_2_O_3_ (100) and (**c**) general representation of Ga-substituted γ-Al_2_O_3_. TEM images of PE particles at different time intervals when the degradation process was performed (**d**) without catalyst, (**e**) with γ-Al_2_O_3_ and (**f**) with Ga-doped γ-Al_2_O_3_.

γ-Al_2_O_3_ effectively catalyzed the PE degradation process and especially enhanced the C-C chain length in the wax produced by PE degradation by inducing ionic character. This is because of the presence of Lewis acid (Al) and Lewis bases (oxygen) in the catalyst. Figure 5a,b are schematic illustrations of the attachment of carbon molecules on γ-Al_2_O_3_. Al acting as a Lewis acid attaches to carbon atoms and imparts a partial negative charge. Meanwhile, oxygen acting as a Lewis base attaches to another carbon atom and imparts a partial positive charge. Hence, ionic character was induced to the C-C bonds which resulted in the weakening of and efficient cracking of the C-C bonds.

The efficiency of γ-Al_2_O_3_ for the induction of ionic character can be further enhanced by doping with Ga because Ga is more electropositive compared to Al. The poor shielding effect increases the effective nuclear charge that holds “s” electrons tightly and reduces the atomic size. Ga has less atomic radius (135 pm) compared to Al (143 pm), which is responsible for its higher ionization energy (Figure 5c). The higher ionization energy of Ga compared to Al makes it a better Lewis acid. The Lewis acidity of an atom/molecule is directly proportional to the ability of the lowest-occupied molecular orbital (LUMO) to accept electron density from the highest-occupied molecular orbital (HOMO) of a Lewis base. In the case of Ga, LUMO has lower energy (compared to Al) and it is easier to attract electrons from Lewis bases of C-C bonds.

To study the effect of reaction time on the degradation (degradation reaction rate) of PE, the reaction was stopped at three different time intervals: 6, 12 and 18 h (Figure 5d–f). PE particles were separated from the catalyst and products on the basis of their peculiar solubility in the binary solution of chloroform and methanol (*v/v*, 2:1). Separated PE particles were analyzed by TEM (Figure 5d–f). It was determined that even when the catalyst was used, degradation took more than 18 h. However, the degradation process was successfully completed after 24 h and no further PE particles were detected. 

## 4. Conclusions 

A mixture of PE and 1-octadecene with Ga-doped γ-Al_2_O_3_ catalyst was degraded to the precursors of gasoline and diesel oil with a trace amount of wax. Heat treatment of PE at a temperature below 250 °C led to the series of reactions resulting in cracking, dehydrogenation and oxidation to produce a mixture consisting of polycarboxylic acids and saturated and unsaturated hydrocarbons. The degradation products were analyzed by ESI-MS and FTIR. ESI-MS analysis revealed that oil precursors consisted of a mixture of hydrocarbons including oxygen and their carbon number varied from C-6 to C-18. Wax also consisted of complicated carboxylic acids and hydrocarbons, with their carbon number ranging from C-22 to C-58. Polyethylene degradation was performed under mild conditions. Furthermore, cheap catalyst (γ-Al_2_O_3_) and solvent (1-octadecene) were used. γ-Al_2_O_3_ effectively catalyzed the degradation process by weakening the C-C bond by enhancing the C-C chain length by the induced ionic character. Being a stronger Lewis acid, Ga doping into γ-Al_2_O_3_ enhanced the catalytic efficiency of C-C and C-H bond degradation. However, there is a margin of improvement whereby if the carbon chain length of the wax (impurity) is further enhanced, the obtained product will be more useful. 

## Supplementary Materials

The following supporting information can be downloaded at: https://www.mdpi.com/article/10.3390/nano12183122/s1, Figure S1. (a) TEM (h) HRTEM and (c–e) TEM-EDS mapping images of γ-Al_2_O_3_; Figure S2. FTIR analysis of the oil precursors with trace amount of wax produced by degradation of polyethylene and 1-degradation, without any catalyst; Figure S3. ESI-MS analysis of the oil precursors produced by the 1-octadecene degradation, without any catalyst; Figure S4. ESI-MS analysis of the oil precursors produced by the 1-octadecene degradation, catalyzed by γ-Al_2_O_3_; Figure S5. ESI-MS analysis of the oil precursors produced by the 1-octadecene degradation, catalyzed by Ga doped γ-Al_2_O_3_; Figure S6. ESI-MS analysis of the wax produced by the polyethylene degradation, without any catalyst; Figure S7. ESI-MS analysis of the wax produced by the polyethylene degradation, catalyzed by γ-Al_2_O_3_; Figure S8. ESI-MS analysis of the wax produced by the polyethylene degradation, catalyzed by Ga doped γ-Al_2_O_3_; Table S1. List of the products obtained during the polyethylene degradation.
