# Supplementary material for: Significance of Ionic Character Induced by Ga-Doped γ-Al2O3 on Polyethylene Degradation to the Precursors of Gasoline and Diesel Oil with a Trace Amount of Wax
Source: Nanomaterials (Basel). 2022 Sep 9;12(18):3122. doi: 10.3390/nano12183122 (PMC9505615; doi:10.3390/nano12183122)
Supplement: Supplementary file 1 [file nanomaterials-12-03122-s001.zip › nanomaterials-1840037-supplementary.pdf]

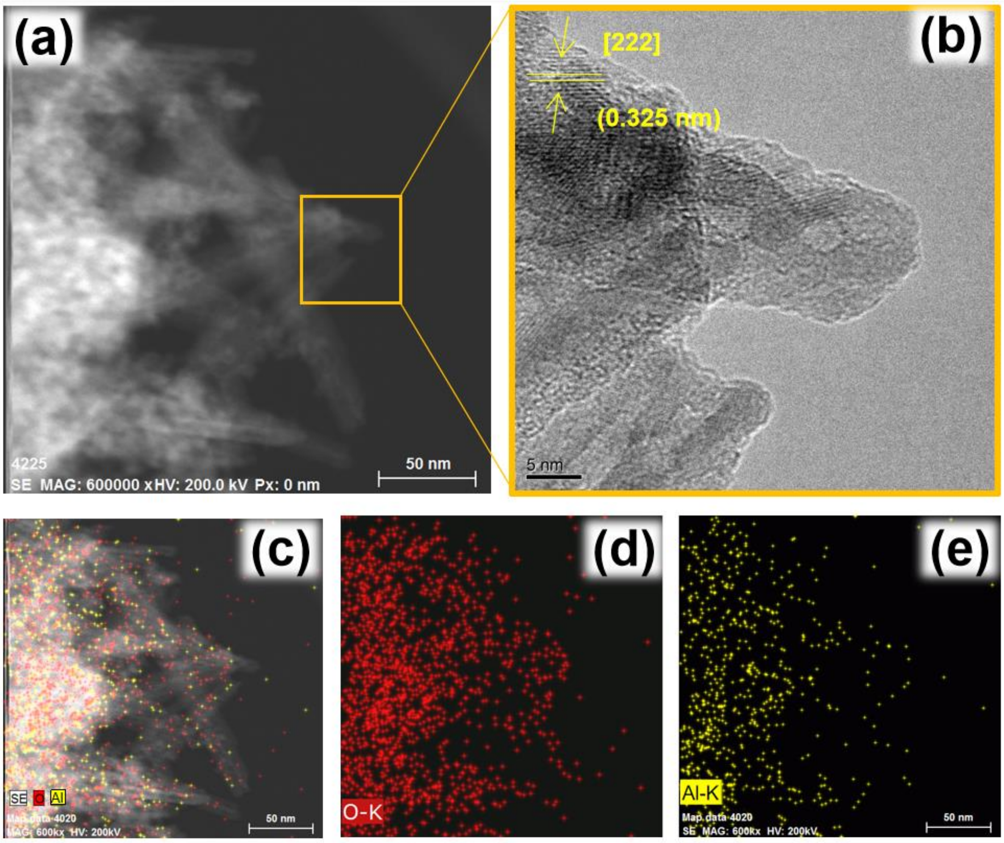


Figure S1. (a) TEM (h) HRTEM and (c-e) TEM-EDS mapping images of γ-$Al_2O_3$

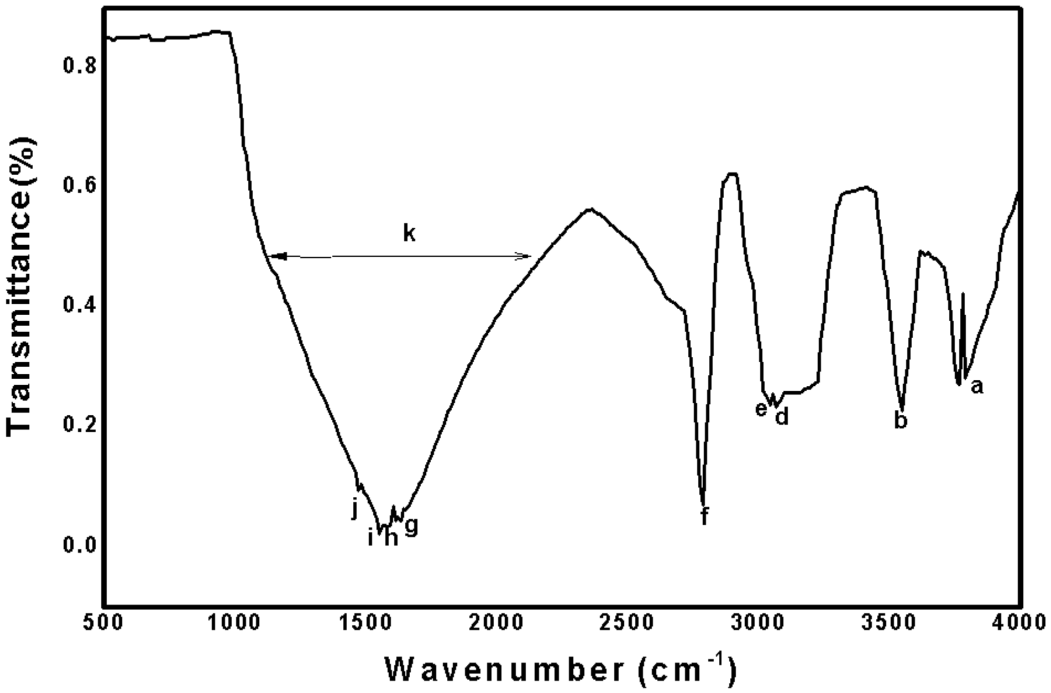


Figure S2. FTIR analysis of the oil precursors with trace amount of wax produced by degradation of polyethylene and 1-degradation, without any catalyst.

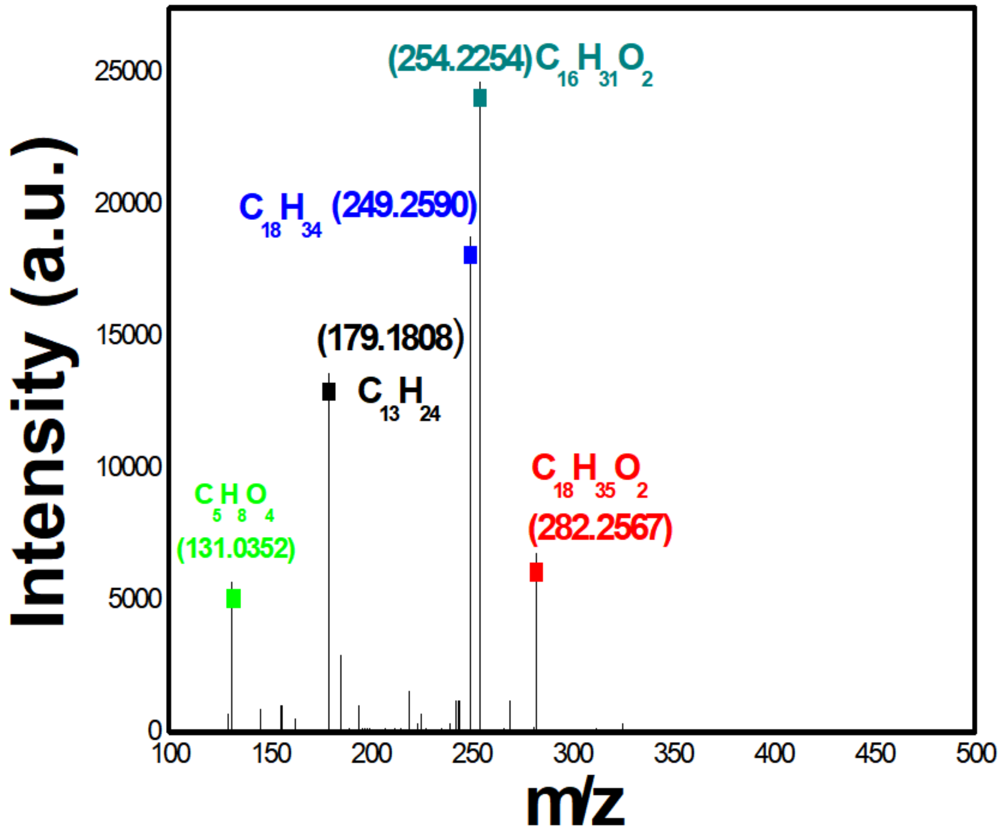


Figure S3. ESI-MS analysis of the oil precursors produced by the 1-octadecene degradation, without any catalyst.

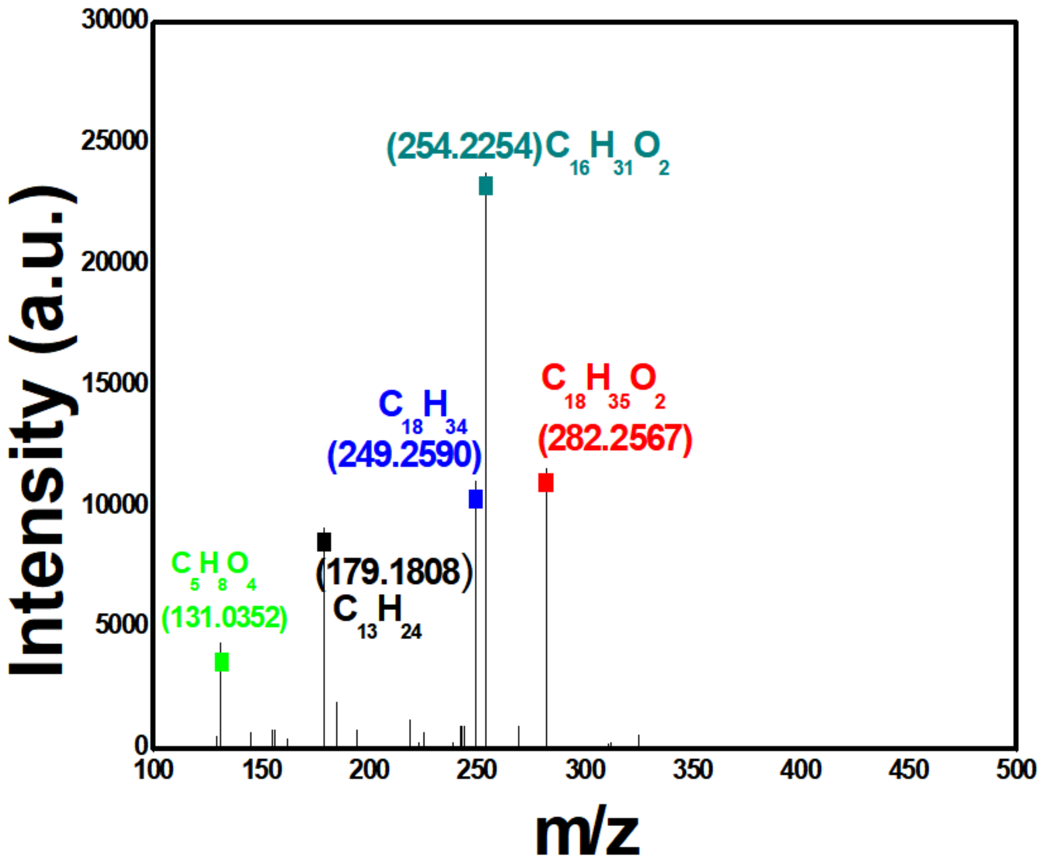


Figure S4. ESI-MS analysis of the oil precursors produced by the 1-octadecene degradation, catalyzed by γ-$Al_2O_3$.

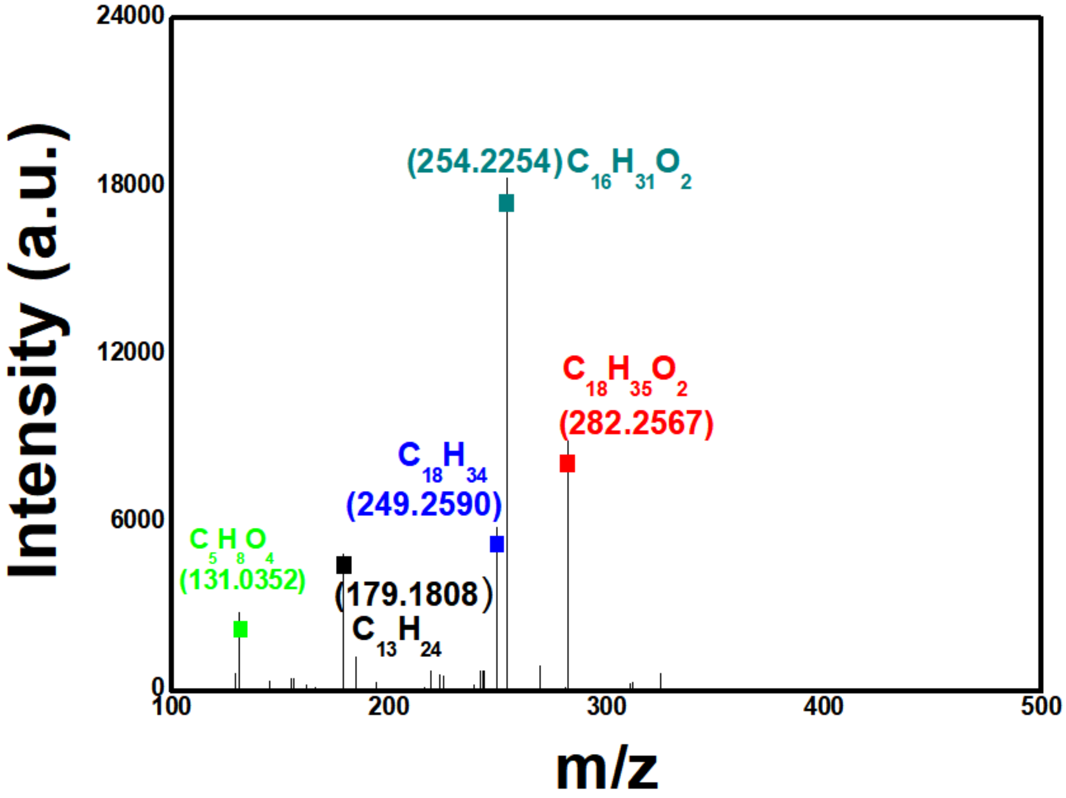


Figure S5. ESI-MS analysis of the oil precursors produced by the 1-octadecene degradation, catalyzed by Ga doped γ-$Al_2O_3$.

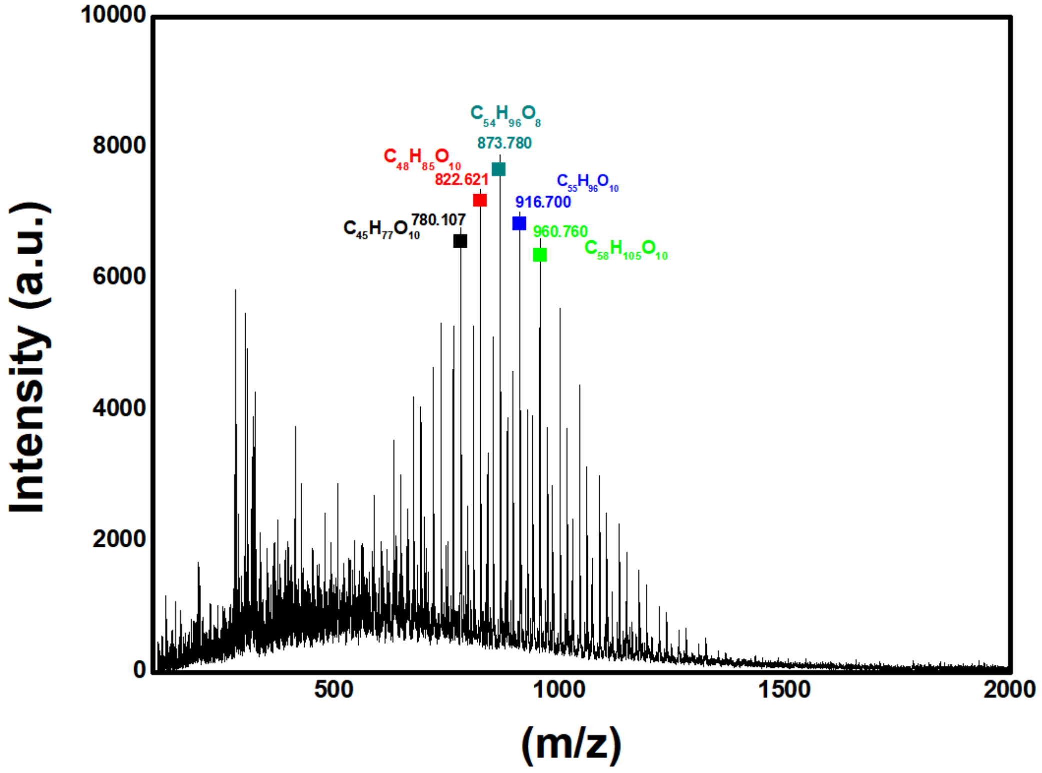


Figure S6. ESI-MS analysis of the wax produced by the polyethylene degradation, without any catalyst.

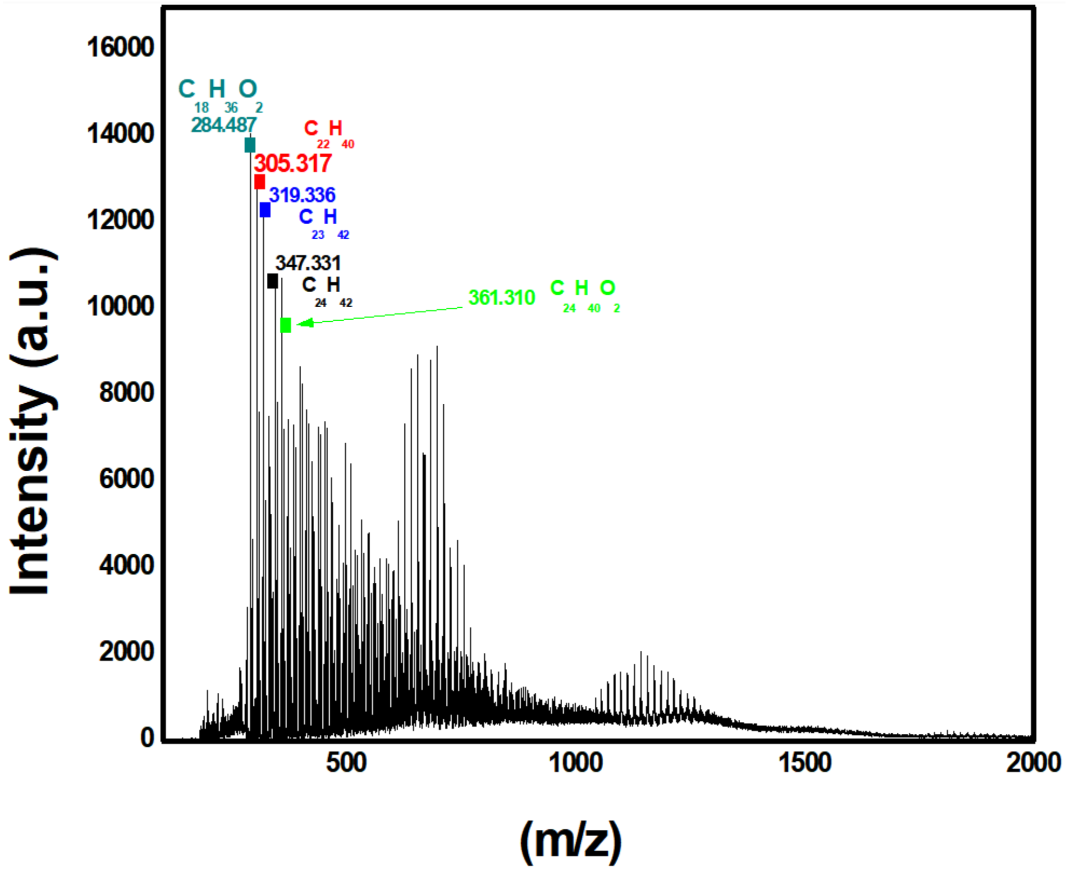


Figure S7. ESI-MS analysis of the wax produced by the polyethylene degradation, catalyzed by γ-$Al_2O_3$.

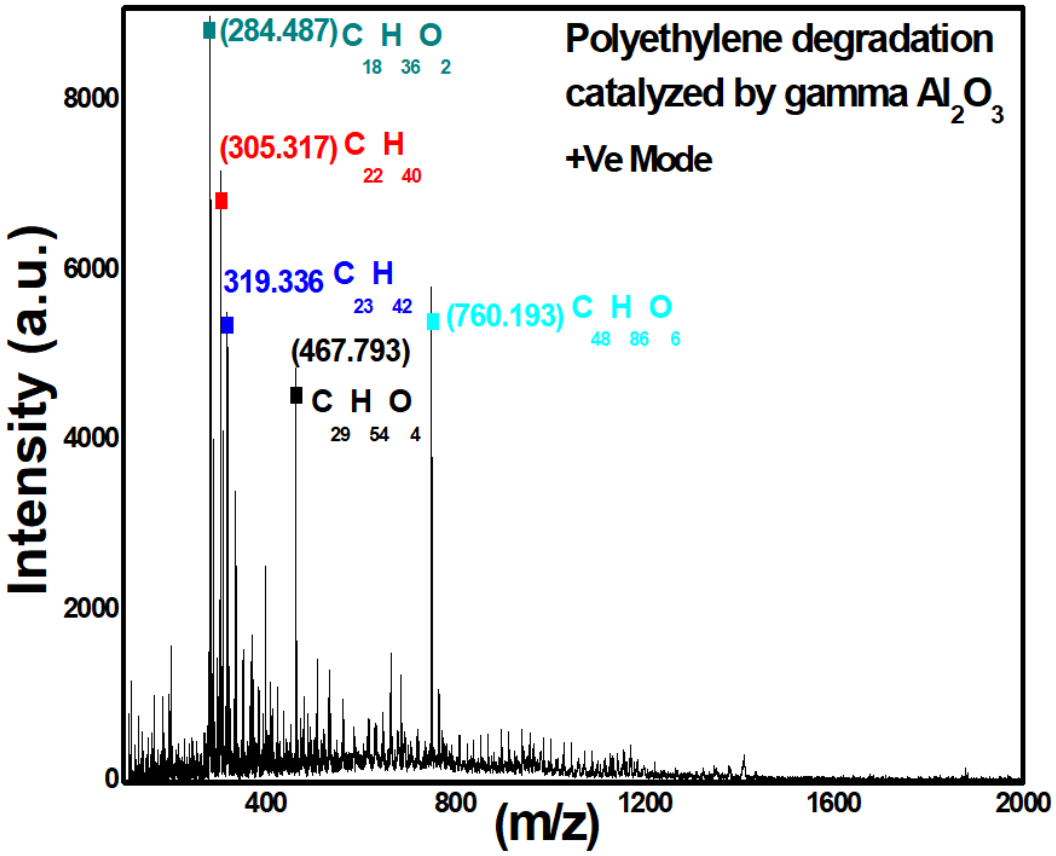


Figure S8. ESI-MS analysis of the wax produced by the polyethylene degradation, catalyzed by Ga doped γ-$Al_2O_3$.

| Degradation Product | [M+H$^+$]* |
|---|---|
| $C_5H_8O_4$ | 131.0352 |
| $C_{13}H_{24}$ | 179.1808 |
| $C_{18}H_{34}$ | 249.259 |
| $C_{16}H_{31}O2$ | 254.2254 |
| $C_{18}H_{35}O_2$ | 282.2567 |
| $C_{22}H_{40}$ | 305.317 |
| $C_{23}H_{42}$ | 319.336 |
| $C_{24}H_{42}$ | 347.331 |
| $C_{24}H_{40}O_2$ | 361.310 |
| $C_{29}H_{54}O_4$ | 467.793 |
| $C_{48}H_{86}O_6$ | 760.193 |
| $C_{54}H_{96}O_8$ | 873.780 |
| $C_{48}H_{85}O_{10}$ | 822.621 |
| $C_{55}H_{96}O_{10}$ | 916.700 |
| $C_{45}H_{77}O_{10}$ | 780.107 |
| $C_{50}H_{88}O_6$ | 785.6651 |
| $C_{52}H_{92}O_7$ | 829.6913 |
| $C_{54}H_{93}O_8$ | 870.694 |
| $C_{54}H_{96}O_8$ | 873.780 |
| $C_{55}H_{96}O_{10}$ | 916.700 |
| $C_{56}H_{96}O_9$ | 913.7124 |
| $C_{58}H_{98}O_{10}$ | 955.723 |
| $C_{58}H_{105}O_{10}$ | 960.760 |

*[M+H+]= Molecular mass+mass of proton

Table S1. List of the products obtained during the polyethylene degradation.
